# Bamboo-inspired optimal design for functionally graded hollow cylinders

**DOI:** 10.1371/journal.pone.0175029

**Published:** 2017-05-03

**Authors:** Motohiro Sato, Akio Inoue, Hiroyuki Shima

**Affiliations:** 1 Division of Engineering and Policy for Sustainable Environment, Faculty of Engineering, Hokkaido University, Sapporo, Hokkaido, Japan; 2 Faculty of Environmental and Symbiotic Sciences, Prefectural University of Kumamoto, Kumamoto, Japan; 3 Department of Environmental Sciences, University of Yamanashi, Kofu, Yamanashi, Japan; Monash University, AUSTRALIA

## Abstract

The optimal distribution of the reinforcing fibers for stiffening hollow cylindrical composites is explored using the linear elasticity theory. The spatial distribution of the vascular bundles in wild bamboo, a nature-designed functionally graded material, is the basis for the design. Our results suggest that wild bamboos maximize their flexural rigidity by optimally regulating the radial gradation of their vascular bundle distribution. This fact provides us with a plant-mimetic design principle that enables the realization of high-stiffness and lightweight cylindrical composites.

## Introduction

Functionally graded materials (FGMs) are a class of advanced materials endowed with spatial variations in the physical properties and/or chemical compositions. The concept of the FGM was first proposed in 1984 [1] for aerospace applications in a spaceplane project. A spaceplane is required to pass repeatedly through the earth’s atmosphere; thus, its body is exposed to very high temperature environments. To endure tremendous heat conditions, researchers had devised a concept for fabricating a material by gradually changing the material composition, as a result of which both the thermal resistance and the mechanical stiffness are improved drastically. Further, the concept of the FGM has been extended to various functionalities beyond the thermal resistance [2, 3]. Currently, various commercial products produced using FGMs can be found in diverse fields including medical devices [4] and energy applications [5].

In addition to artificial FGMs, naturally designed FGMs can be found invariably in our daily life, ranging from bones and teeth in our body to seashells and plants observed outdoors. A possible reason for the frequent realization of FGMs in nature is that the biological cells tend to adapt to external stimuli; their self-adaptive property may have caused a naturally gradient structure during the evolutionary process for enhancing the stiffness-to-weight ratio.

**Fig 1:**
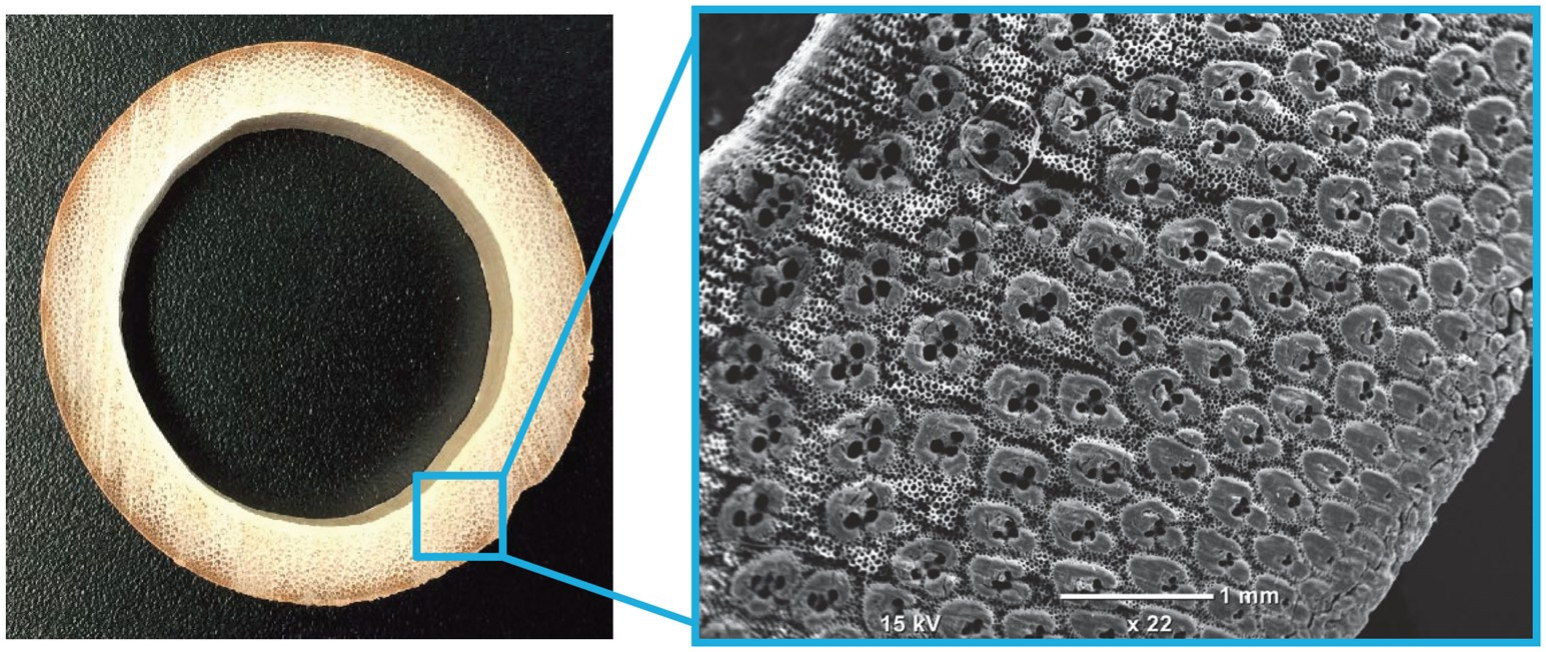
Photo of bamboo’s cross section. A wild moso bamboo (*Phyllostachys pubescens*) was sampled and its cross section was observed with a microscope. The gradient distribution of the vascular bundles across the wall thickness is clearly observed.

Amongst several candidates, bamboo is one of the most typical and intriguing FGMs that occurs in nature [6–9]. Most bamboos are cylindrical hollow plants native to all continents except Antarctica and Europe [10]. From a structural perspective, bamboo is a fiber-reinforced composite material made of two main types of tissues: vascular bundles as the reinforcing fibers and a matrix of parenchyma cells [11–13]. These fibers are sufficiently rigid; hence, their Young’s modulus is in the order of several gigapascals, comparable to that of steel [13, 14]. The fibers are aligned in the longitudinal direction, being embedded in a continuous host matrix that does not contribute much to the rigidity of bamboo [12]. Bamboo is a nature-designed FGM because the volume fraction of the fibers increases radially from the inner to the outer surface of the bamboo culm [11, 15–23] (See Fig 1). The biased distribution of the fibers across the wall thickness is considered to enhance the flexural rigidity of the culm as a whole against crosswinds and bending because of gravity [15, 19]. A closer look at the fiber distribution in the cross section, therefore, will lead us to an optimal design strategy for fiber-reinforced cylindrical composites inspired by the bamboo morphology [24–26].

In the present work, we have uncovered the strategy by which wild bamboo optimizes the radial distribution of the vascular bundles in the cross section. By imposing a few realistic assumptions, we have formulated the flexural rigidity of the fiber-reinforced cylindrical composites and identified the methodology of regulating the fiber distribution for maximizing the flexural rigidity. Surprisingly, the optimal fiber distribution that we have theoretical deduced is in quantitative agreement with the earlier experimental data of the vascular bundle distribution in wild moso bamboo. This agreement implies a self-optimizing property in the wild bamboo’s structure and provides us with an optimal design principle for the artificial cylindrical composites.

## Volume fraction of the fiber

**Fig 2:**
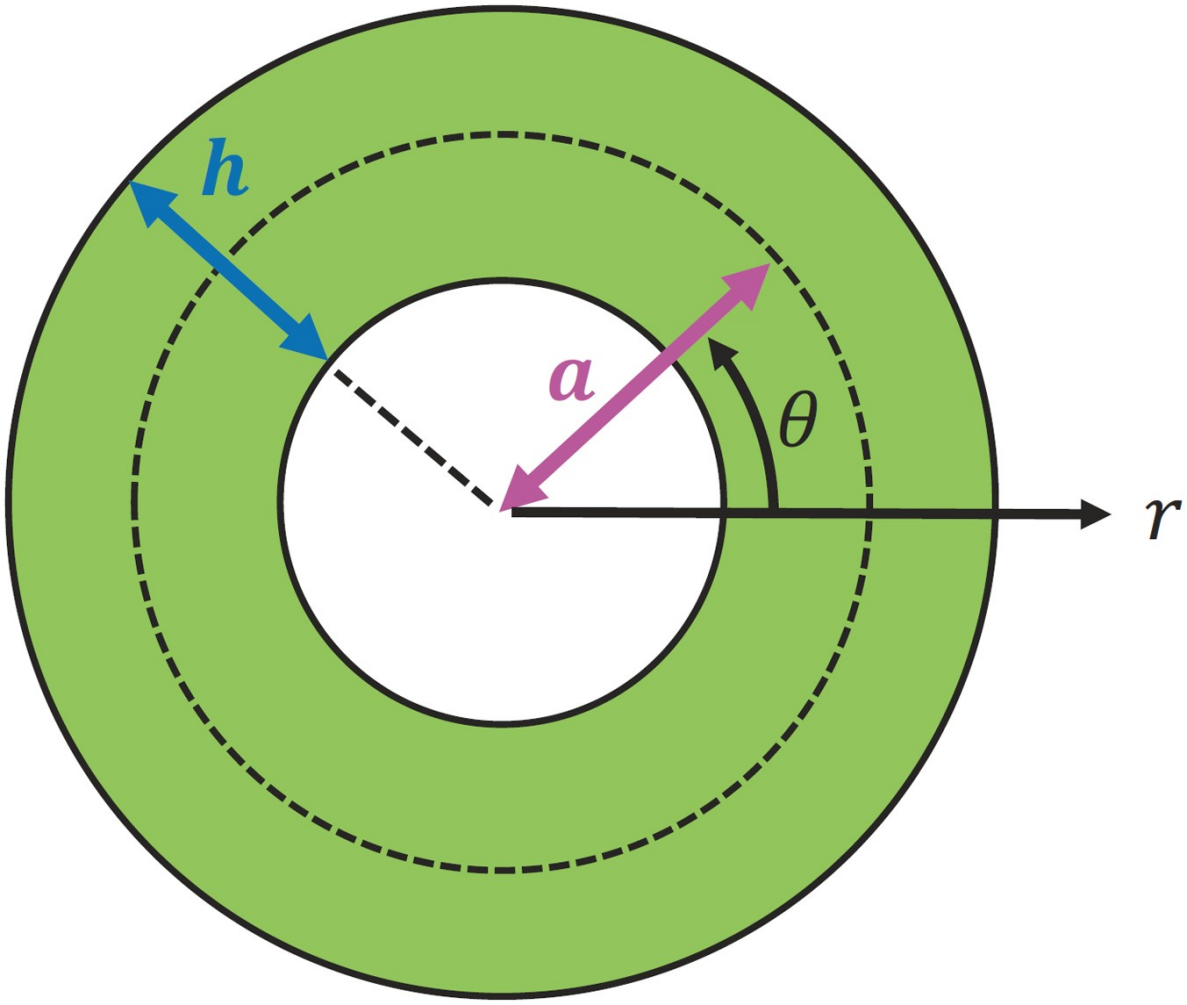
Theoretical model of hollow cylinder’s section. The cross section of a hollow cylinder that mimics wild bamboo is depicted. The definitions of the geometric parameters, *a* and *h*, and the radial coordinates, *r* and *θ*, used in the analysis are presented.

We model a bamboo culm as an elastic hollow cylinder with a radius, *a*, and a wall thickness, *h*. Refer Fig 2 for a diagram of the cylinder’s cross section. Over the section, the reinforcing fibers are scattered in a gradient from the inner hollow core to the outer surface in a radial direction. Our central hypothesis is that the volume fraction of the fibers, denoted by *V*_*f*_(*r*), follows the expression below:

$$\begin{array}{c} V_{f}\left(r\right)=c_{0}+c_{1}r+c_{2}r^{2}\;\left(a-\frac{h}{2}\leq r\leq a+\frac{h}{2}\right). \end{array}$$ (1)

The assumption made in Eq (1) is based on a series of experimental measurements. An earlier work has suggested that the measured data, *V*_*f*_(*r*), for the real bamboo can be well fitted by the curve of a gradually increasing function with respect to *r*. The precise form of the curve was found to be either linear [27], parabolic [6, 17], or other smooth curves [16, 23, 28], depending upon the bamboo species and the height of the culm section from the ground. Note that Eq (1) does not rule out the possibility that *V*_*f*_(*r*) may be linear with respect to *r*, as the linearity is restored by setting, *c*_2_ ≡ 0.

In a real bamboo’s cross section, a limited (but large) number of vascular bundles are distributed optimally for maximizing the flexural rigidity of the entire culm. To explore the optimal distribution, we start from a virtual situation, wherein a given number of reinforcing fibers are uniformly scattered with equal density throughout the wall thickness; we then impose a spatial gradient to the fiber’s density, while maintaining the total number of fibers unchanged. In the initial virtual situation, *V*_*f*_(*r*) is a constant, denoted by $V_{f}^{\text{av}}$, at arbitrary positions in the cross section. After imposing a certain gradient, *V*_*f*_(*r*) becomes *r*-dependent. The effect of the gradient distribution is thus evaluated by comparing the flexural rigidity obtained from the *r*-dependent *V*_*f*_(*r*) with that from the constant $V_{f}^{\text{av}}$.

In tuning the degree of the spatial gradient imposed on the fiber density, we assume that the total number of vascular bundles is maintained at a constant. The hypothesis requires *V*_*f*_(*r*) to follow the relationship:

$$\begin{array}{c} 2\pi ah\cdot V_{f}^{\text{av}}=\int_{0}^{2\pi }d\theta \int_{a-\frac{h}{2}}^{a+\frac{h}{2}}V_{f}\left(r\right)rdr. \end{array}$$ (2)

The left side of Eq (2) represents the total number of vascular bundles that should be conserved. Substituting Eq (1) into the above, the conservation hypothesis is expressed in terms of the coefficients, *c*_*j*_, as:

$$\begin{array}{c} V_{f}^{\text{av}}=\frac{1}{ah}\sum_{j=0}^{2}\frac{c_{j}}{j+2}\left[{\left(a+\frac{h}{2}\right)}^{j+2}-{\left(a-\frac{h}{2}\right)}^{j+2}\right]. \end{array}$$ (3)

Eq (3) indicates that one of the three coefficients, {*c*_*j*_} (*j* = 0, 1, 2), should be dependent on the remaining two. We choose *c*_0_ as the dependent variable and rewrite Eq (3) as:

$$\begin{array}{c} c_{0}=V_{f}^{\text{av}}-\left(a+\frac{h^{2}}{12a}\right)c_{1}-\left[a^{2}+{\left(\frac{h}{2}\right)}^{2}\right]c_{2}. \end{array}$$ (4)

We further impose the following assumptions:

1. *V*_*f*_(*r*) monotonically increases with respect to *r*; particularly, when *c*_2_ ≠ 0, it is a downward convex.
2. *V*_*f*_(*r*) at the outer surface is suppressed by an upper limit denoted by $V_{f}^{\text{out}}$.
3. *V*_*f*_(*r*) at the inner surface does not fall below a lower limit denoted by $V_{f}^{\text{inn}}$.

The first of the three assumptions is based on experimental facts as mentioned earlier [6, 17, 27]. Using mathematical expressions, it is represented as:

$$\begin{array}{c} c_{2}\geq 0\;\text{and}\;\frac{c_{1}}{2c_{2}}\geq -\left(a-\frac{h}{2}\right). \end{array}$$ (5)

The latter two assumptions are represented as:

$$\begin{array}{c} c_{0}+c_{1}\left(a+\frac{h}{2}\right)+c_{2}{\left(a+\frac{h}{2}\right)}^{2}\leq V_{f}^{\text{out}}, \end{array}$$ (6)

and

$$\begin{array}{c} c_{0}+c_{1}\left(a-\frac{h}{2}\right)+c_{2}{\left(a-\frac{h}{2}\right)}^{2}\geq V_{f}^{\text{inn}}. \end{array}$$ (7)

For the wild moso bamboo (*Phyllostachys pubescens*), for example, $V_{f}^{\text{out}}$ and $V_{f}^{\text{inn}}$ were found to have values of ca. 0.6 and 0.1, respectively [6, 15–17, 23, 27]. We will use these values in the subsequent discussion without loss of generality, because the conclusion obtained does not significantly depend on their numerical settings.

It should be emphasized that the value of *c*_0_ cannot be set freely but is uniquely determined by those of *c*_1_ and *c*_2_, through Eq (4). As a consequence, the set of four inequalities Eqs (5)–(7) specifies a closed polygonal domain in the *c*_1_-*c*_2_ coordinate space. From the domain, we will seek a pair of coefficients, (*c*_1_, *c*_2_), that maximize the flexural rigidity of the hollow cylinder.

## Flexural rigidity

We are in a position to derive the flexural rigidity, *D*_*c*_, of the fiber-reinforced cylindrical composites under bending. It follows from the definition:

$$\begin{array}{c} D_{c}=\int_{a-\frac{h}{2}}^{a+\frac{h}{2}}dr\int_{0}^{2\pi }d\theta \left[E_{c}\left(r\right)\cdot r^{3}\sin^{2}\theta \right]. \end{array}$$ (8)

Here, *E*_*c*_(*r*) represents the *r*-dependent local Young’s modulus of the cylindrical composite. In the present situation, it is a linear combination of the Young’s modulus of the reinforcing fiber, *E*_*f*_ and that of the host matrix, *E*_*m*_, expressed as:

$$\begin{array}{c} E_{c}\left(r\right)=E_{f}V_{f}\left(r\right)+E_{m}\left[1-V_{f}\left(r\right)\right]. \end{array}$$ (9)

Substituting Eqs (1) and (9) in Eq (8), we can express *D*_*c*_ as a quadratic form with respect to *c*_*j*_. Similarly, the counterpart for the virtual case of the constant fiber distribution, $D_{c}^{0}$, is given by: $D_{c}^{0}=\int_{a-(h/2)}^{a+(h/2)}dr\int_{0}^{2\pi }d\theta \left[E_{c}^{0}\cdot r^{3}\sin^{2}\theta \right]$. Thus, we have obtained the explicit forms of the flexural rigidity under two different conditions:

$$\begin{array}{c} D_{c}=\pi \left(E_{f}\;-\;E_{m}\right)\cdot \left(\sum_{j=0}^{2}\frac{c_{j}}{j+4}\cdot A_{j+4}\right)+\;B, \end{array}$$ (10)

$$\begin{array}{c} D_{c}^{0}=\pi \left(E_{f}-E_{m}\right)\left[a^{2}\;\;+{\left(\frac{h}{2}\right)}^{2}\right]\left(\sum_{j=0}^{2}\frac{c_{j}}{j+2}A_{j+2}\right)\;\;+\;B, \end{array}$$ (11)

with

$$\begin{array}{c} A_{n}\equiv {\left(a+\frac{h}{2}\right)}^{n}-{\left(a-\frac{h}{2}\right)}^{n},\;\;B\equiv \pi E_{m}\cdot ah\left[a^{2}+{\left(\frac{h}{2}\right)}^{2}\right]. \end{array}$$ (12)

It is to be noted again that *c*_0_ is not an independent variable but a linear combination of *c*_1_ and *c*_2_. As a consequence, both *D*_*c*_ and $D_{c}^{0}$ are reduced to quadratic forms of *c*_1_ and *c*_2_. Our remaining task is, therefore, to find the optimal values of *c*_1_ and *c*_2_ that maximize *D*_*c*_ for a given set of parameters: $V_{f}^{\text{av}}$, *E*_*f*_, *E*_*m*_, *a*, and *h*. The effect of the functional gradient on the enhancement in *D*_*c*_ is quantified by,

$$\begin{array}{c} \eta \equiv \frac{D_{c}-D_{c}^{0}}{D_{c}^{0}}. \end{array}$$ (13)

Note that the last terms in the right sides of both the Eqs (10) and (11) do not contribute to *η*, as they are identical to each other.

## Variable transformation

In the subsequent discussion, we normalize the radial coordinate, *r*, to $\bar{r}$ so that $\bar{r}=0$ at the innermost and $\bar{r}=1$ at the outermost surface. Using mathematical expressions, we transform *r* to $\bar{r}$:

$$\begin{array}{c} \bar{r}=\frac{1}{h}\left[r-\left(a-\frac{h}{2}\right)\right], \end{array}$$ (14)

that results in,

$$\begin{array}{c} V_{f}\left(\bar{r}\right)={\bar{c}}_{0}+{\bar{c}}_{1}\bar{r}+{\bar{c}}_{2}{\bar{r}}^{2},\;\left(\;0\leq \bar{r}\leq 1\;\right) \end{array}$$ (15)

with the following coefficients:

$$\begin{array}{ccc} {\bar{c}}_{0} & = & c_{0}+c_{1}\left(a-\frac{h}{2}\right)+c_{2}{\left(a-\frac{h}{2}\right)}^{2}, \end{array}$$ (16)

$$\begin{array}{ccc} {\bar{c}}_{1} & = & c_{1}h+c_{2}\cdot 2h\left(a-\frac{h}{2}\right), \end{array}$$ (17)

$$\begin{array}{ccc} {\bar{c}}_{2} & = & c_{2}h^{2}. \end{array}$$ (18)

Again, we assume that ${\bar{c}}_{0}$ is dependent on the remaining two; in addition, the four inequalities given by Eqs (5)–(7) are rewritten in terms of $\left\{{\bar{c}}_{j}\right\}$ (*j* = 0, 1, 2), specifying a closed polygonal domain in the ${\bar{c}}_{1}$-${\bar{c}}_{2}$ space.

## Results and discussion

### Maximal condition for the flexural rigidity

**Fig 3:**
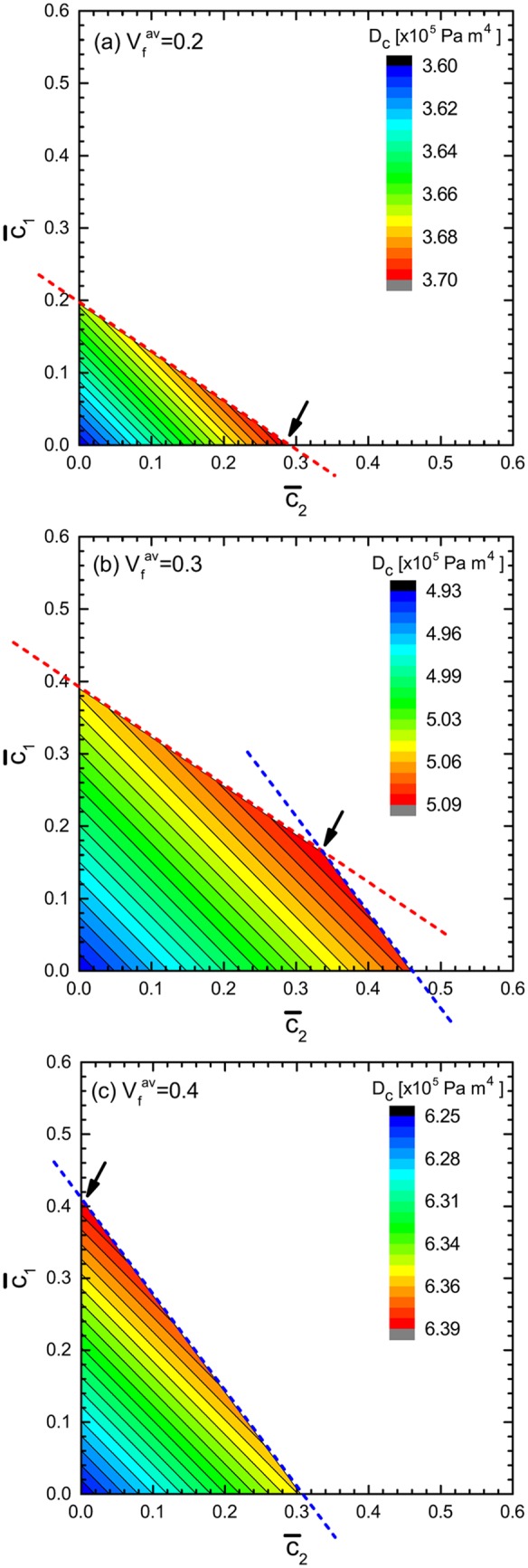
Contour plot of flexural rigidity. The flexural rigidity, *D*_*c*_, of the cylindrical FGMs for different values of $V_{f}^{\text{av}}$ is presented. The arrows indicate the maximal point of *D*_*c*_ in the ${\bar{c}}_{1}$-${\bar{c}}_{2}$ space. See text for the details of the parameter settings.

Fig 3 shows the contour plots of the flexural rigidity, *D*_*c*_, in the ${\bar{c}}_{2}$-${\bar{c}}_{1}$ space; the values of *D*_*c*_ are in the unit, Pa ⋅ m^4^. From panels (a) to (c), $V_{f}^{\text{av}}$ is set to increase from 0.2 to 0.4 as indicated in the legend. For all the plots, we fixed the radius to be *a* = 10.0 cm and the wall thickness to be *h* = 1.5 cm, referring to the typical geometry of the real moso bamboos [29]. In addition, we set *E*_*f*_ = 30 GPa and *E*_*m*_ = 2 GPa also referring to moso bamboo [14, 16, 17, 21, 23, 27, 30]. $V_{f}^{\text{out}}$ and $V_{f}^{\text{inn}}$ are fixed to be 0.6 and 0.1, respectively [6, 15–17, 23, 27].

In all the three panels, the magnitude of *D*_*c*_ increases monotonically from the bottom left to the upper right in the ${\bar{c}}_{2}$-${\bar{c}}_{1}$ space. The increase is however, truncated by one or a pair of boundary lines depicted by slanted dashed lines in the panels. The dashed line in red originates from the inequality of Eq (7), imposing the restriction that *V*_*f*_ at the inner surface (*i.e.,* $\bar{r}=0$) must be larger than $V_{f}^{\text{inn}}$. Similarly, the boundary line in blue is caused by the inequality of Eq (6), requiring that *V*_*f*_ at the outer surface (*i.e.,* $\bar{r}=1$) must not exceed $V_{f}^{\text{out}}$. As a consequence, the two slanted lines set a closed domain (square or triangle) in the first quadrant of the ${\bar{c}}_{2}$-${\bar{c}}_{1}$ space. The area and shape of the domain depend upon the value of $V_{f}^{\text{av}}$, as illustrated in Fig 3 because an increase in $V_{f}^{\text{av}}$ causes the red (blue) slanted line to move upward (to the right side), while retaining its slope.

An important observation in Fig 3 is that the relative configuration of the two slanted lines determines the position in the ${\bar{c}}_{2}$-${\bar{c}}_{1}$ space that maximizes *D*_*c*_. In the case of Fig 3(a), for instance, the blue line is located at the far right from the origin, thus, it does not intersect with the red line in the first quadrant of the ${\bar{c}}_{2}$-${\bar{c}}_{1}$ space. As a result, *D*_*c*_ becomes maximum at the point, $\left({\bar{c}}_{2},{\bar{c}}_{1}\right)=\left(0.29,0\right)$, namely, the intercept of the red line with respect to the ${\bar{c}}_{2}$ axis. A parallel argument holds for the case of Fig 3(c), in which the red line shifts far above; thus, *D*_*c*_ becomes maximum at the intercept of the blue line with respect to the ${\bar{c}}_{1}$ axis. An intermediate case is presented in Fig 3(b); the two slanted lines cross within the first quadrant of the space and the crossing point corresponds to the maximal point of *D*_*c*_. Accordingly, the maximal point of *D*_*c*_ moves from the right bottom [see panel (a)] to the left top [see panel (c)] on increasing $V_{f}^{\text{av}}$.

The result mentioned above suggests a preferred strategy for the spatial gradient in the fiber density. If $V_{f}^{\text{av}}$ is relatively small, a parabolic distribution (*i.e.,* *c*_1_ ∼ 0) is preferred for enhancing the *D*_*c*_ of the cylindrical composites. This is the case when the total number of reinforcing fibers is less and/or the fibers are considerably thin. Otherwise, if $V_{f}^{\text{av}}$ is larger, a linear distribution (*i.e.,* *c*_2_ ∼ 0) is preferred for enhancing *D*_*c*_. We also confirmed that a similar trend in the correlation between the preferred fiber distribution and the magnitude of $V_{f}^{\text{av}}$ was observed in most of the various parameter conditions for $V_{f}^{\text{out}}$ and $V_{f}^{\text{inn}}$, as well as, *a* and *h*, even though the degree of enhancement of *D*_*c*_ depends upon the choices of the parameter values.

### Variation in the optimal coefficients

**Fig 4:**
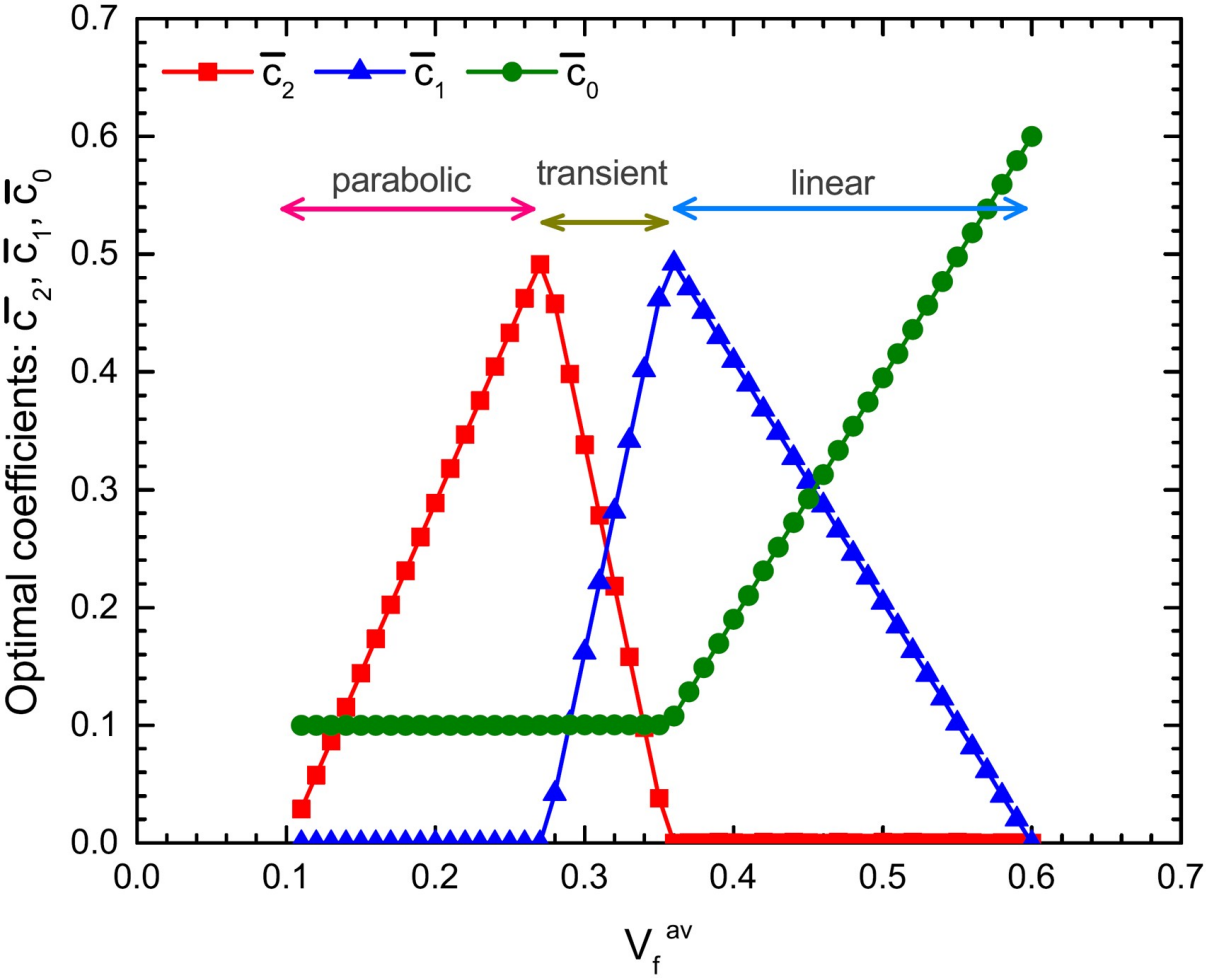
Diagram of optimal coefficient. $V_{f}^{\text{av}}$-dependence of the optimal coefficients: ${\bar{c}}_{2},{\bar{c}}_{1},{\bar{c}}_{0}$ is presented. A “parabolic” (or “linear”) zone implies that for a given $V_{f}^{\text{av}}$, the optimal curve of $V_{f}\left(\bar{r}\right)$ satisfies ${\bar{c}}_{1}=0$ (${\bar{c}}_{2}=0$).

Fig 4 summarizes the systematic variation in the optimal coefficients, ${\bar{c}}_{j}$ (*j* = 0, 1, 2), associated with the change in $V_{f}^{\text{av}}$. The material parameters, $V_{f}^{\text{out}}$ and $V_{f}^{\text{inn}}$, as well as the geometric parameters, *a* and *h*, are all set to be identical to those used in Fig 3. The diagram shows that for $V_{f}^{\text{av}}<0.27$, *D*_*c*_ is maximized by setting a parabolic distribution, $V_{f}\left(\bar{r}\right)={\bar{c}}_{0}+{\bar{c}}_{2}{\bar{r}}^{2}$, with specific values of ${\bar{c}}_{0}$ and ${\bar{c}}_{2}$. On the other hand, for $V_{f}^{\text{av}}>0.36$, it is maximized by a linear distribution, $V_{f}\left(\bar{r}\right)={\bar{c}}_{0}+{\bar{c}}_{1}\bar{r}$, with specific values of ${\bar{c}}_{0}$ and ${\bar{c}}_{1}$. In-between $V_{f}^{\text{av}}$, all the ${\bar{c}}_{j}$ (*j* = 0, 1, 2) take nonzero values. To depict an overall behavior, we introduced the concept of the three characteristic zones as shown in Fig 4. A “parabolic” (or “linear”) zone implies that for a given $V_{f}^{\text{av}}$, the optimal radial distribution is given by a parabolic curve (linear straight line) of $V_{f}\left(\bar{r}\right)$ characterized by ${\bar{c}}_{1}=0$ (${\bar{c}}_{2}=0$). The two zones are separated by a “transient” zone in which both ${\bar{c}}_{2}$ and ${\bar{c}}_{1}$ provide certain contributions to the curve, $V_{f}\left(\bar{r}\right)$.

We have numerically checked that the conclusion holds for almost all the cylinder geometries (*i.e.,* for all the selected values of *a* and *h*), while the precise locations of the zone boundaries and zone widths are dependent on the choices of the parameter values. For instance, if we set *a* = 10.0 cm and *h* = 10.0 cm, without changing the values of $V_{f}^{\text{out}}$ and $V_{f}^{\text{inn}}$, the three line charts of ${\bar{c}}_{2}$, ${\bar{c}}_{1}$, and ${\bar{c}}_{0}$ shown in Fig 4 move collectively to the right. As a consequence, the parabolic-transient (p-t) zone boundary shifts to $V_{f}^{\text{av}}=0.31$ and the transient-linear (t-l) zone boundary shifts to $V_{f}^{\text{av}}=0.39$. In contrast, if we set *a* = 10.0 cm and *h* = 0.3 cm, the zone boundaries of p-t and t-l shift very slightly to the left to $V_{f}^{\text{av}}=0.27$ and $V_{f}^{\text{av}}=0.35$, respectively. Despite the translational shift of the zone boundaries, the overall profiles of the three line charts, (*i.e.,* the combination of the two saw-tooth and the initially horizontal one, followed by a diagonal increase up to the right) are considerably robust against the variations in the value of *h*/*a*.

It is to be further noted that the diagram shown in Fig 4 is completely invariant to the change in *h* and *a*, if the ratio *h*/*a* is maintained constant. This invariance facilitates the efficiency of our theoretical approach in describing the mechanics of the wild bamboo. It is widely accepted that in wild bamboo, the ratio of the two sectional areas, explained below as i) and ii), is constant throughout an individual bamboo culm [29]: i) the sectional area of the culm’s woody portion, *i.e.,* 2*πah* deduced from Fig 1; ii) the sectional area of the circle enclosed by the outer surface, *i.e.,* *π*[*a*+(*h*/2)]^2^ from Fig 1. The constant ratio throughout the culm implies that the thickness-radius ratio (*i.e.,* *h*/*a*) of the wild bamboo also has an identical value throughout an individual bamboo culm from the ground to the top [31]. In the case of the moso bamboo, for example, it has a value, *h*/*a* ∼ 0.15, irrespective of the height above the ground level of the section, although the values of *a* and *h* are significantly dependent on the height. The quantitative uniqueness of *h*/*a* throughout a given bamboo culm and the robustness in the diagram shown in Fig 4 for a given value of *h*/*a* indicate that the diagram applies to culm sections at arbitrary heights from the ground.

### Optimal fiber distribution

**Fig 5:**
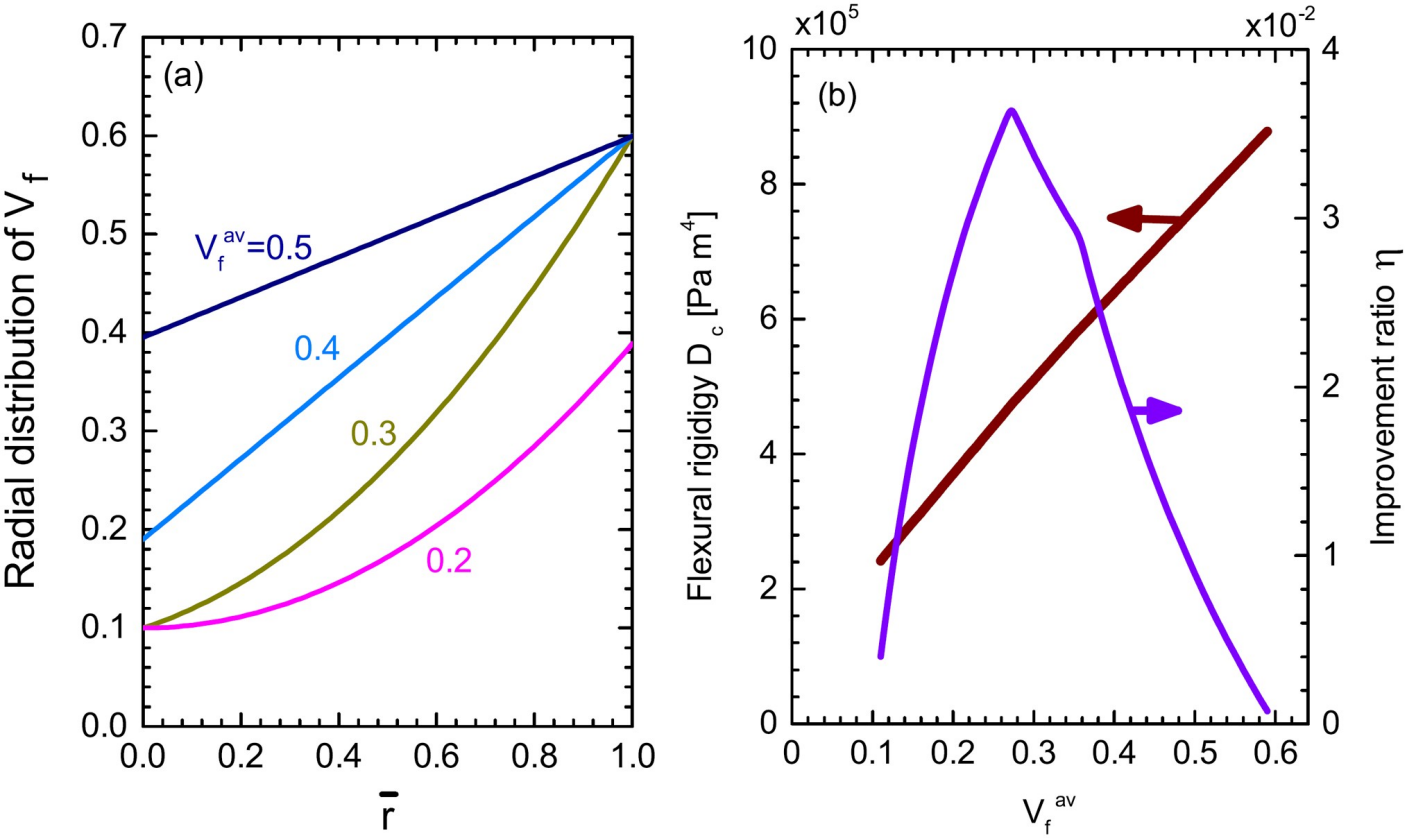
Effects of tuning fiber volume fraction. (a) Optimal fiber distribution, $V_{f}\left(\bar{r}\right)$, for various conditions of $V_{f}^{\text{av}}$. (b) Maximized flexural rigidity, *D*_*c*_ and the improvement ratio in the rigidity, *η*.

Fig 5(a) illustrates the evolution of the optimal fiber distribution curve on increasing $V_{f}^{\text{av}}$. The same parameter values as those in Figs 3 and 4 are used. The remarkable fact is that the $V_{f}^{\text{av}}$-driven change in the profile of the optimal $V_{f}\left(\bar{r}\right)$ curve shown in Fig 5(a), is in quantitative agreement with the experimental data on the vascular bundle distribution within the wild moso bamboo. It was observed in Ref. [17] that in the wild moso bamboo, the best fitting curve for the radial distribution data of the vascular bundles varies gradually from parabolic to linear on changing the height of sampled culm sections. Namely, the section near the ground showed a relatively small $V_{f}^{\text{av}}$ (ca. 0.2), causing a parabolic distribution of the vascular bundles over the section. On the other hand, the section close to the top showed a larger $V_{f}^{\text{av}}$ (ca. 0.4), resulting in a linear distribution. These experimental observations are well reproduced by the theory that we have developed, as clearly proved in Fig 5(a); It is also reminiscent of the measurement reported in Ref. [17]. The consistency between the theory and the experiment supports the validity of our hypothesis that wild bamboos regulate the spatial distribution of the vascular bundles within a section, thereby, maximizing their flexural rigidity.

Fig 5(b) shows a monotonic growth in *D*_*c*_ with an increase in $V_{f}^{\text{av}}$; it also plots the $V_{f}^{\text{av}}$ dependence of *η*, the improvement ratio in the flexural rigidity induced by the gradient distribution of the fibers. The magnitude of *η* is maximized at $V_{f}^{\text{av}}=0.28$ under the present condition, coinciding with the zone boundary that separates the parabolic and transient zones (see Fig 4). In this *η*-maximal situation, the optimal distribution curve satisfies $V_{f}\left(\bar{r}\right)=V_{f}^{\text{inn}}$ at $\bar{r}=0$, $V_{f}\left(\bar{r}\right)=V_{f}^{\text{out}}$ at $\bar{r}=1$, and the degree of downward convexity in the curve of $V_{f}\left(\bar{r}\right)$ is as large as possible. Beyond the *η*-maximal situation, the degree of convexity is allowed to degrade on increasing $V_{f}^{\text{av}}$ in order to maximize *D*_*c*_, although *η* itself decreases, as observed in Fig 5(b). This causes a crossover from the parabolic zone to the linear zone, as shown in Figs 4 and 5(a).

### Comparison with experimental data

**Fig 6:**
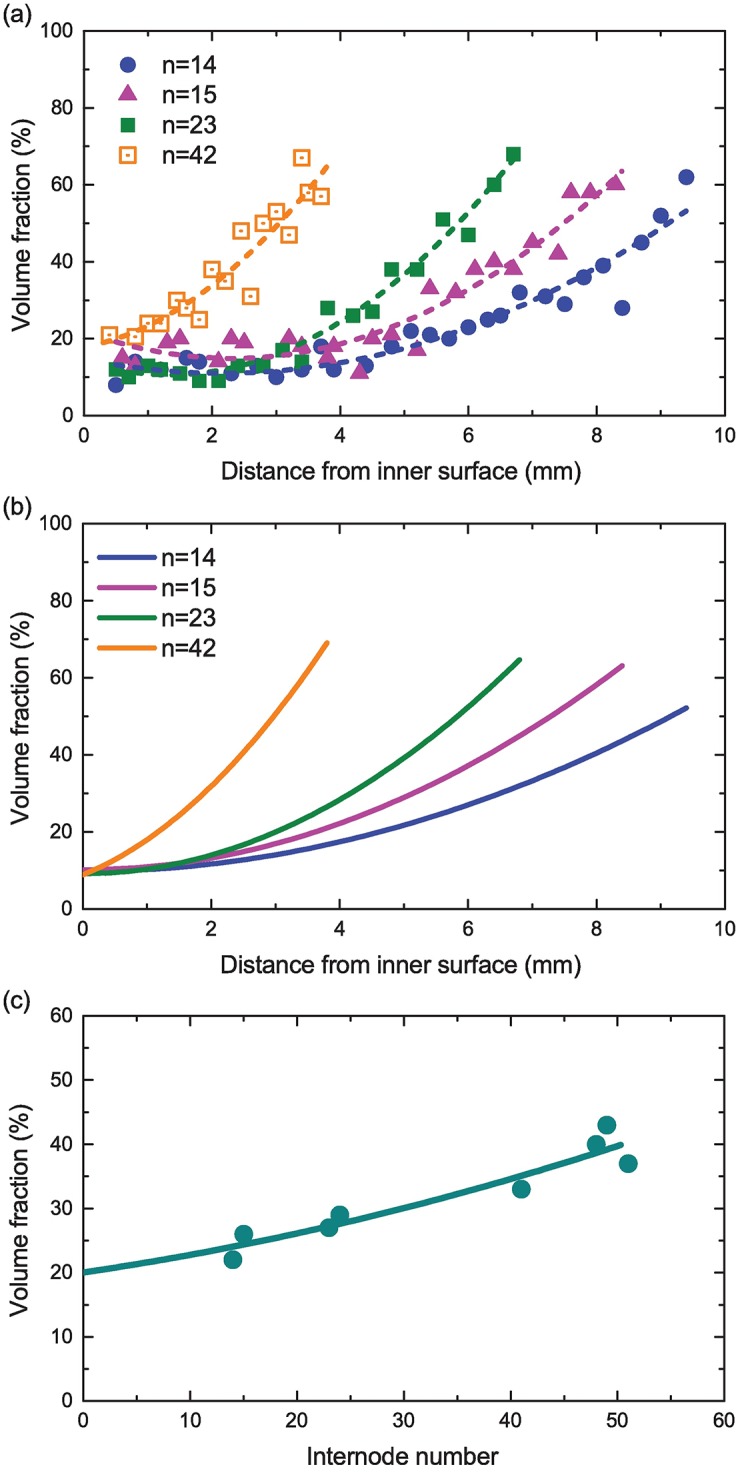
Theory-experiment comparison. (a) Experimentally measured values of the fiber’s volume fraction in wild moso bamboos as a function of the distance from the inner surface of the hollow culm. The index *n* in the legend represents the internode number from the ground. The original graph was presented in Ref. [17]. (b) Theoretically-derived optimal distribution of fibers in elastic hollow cylinders. (c) Experimentally observed relation of the fiber volume fraction with the internode number. The original graph was given in Ref. [17].

A direct comparison of our theoretical results for the optimal fiber distribution with the existing experimental data for wild bamboos assures the validity of the theory we have developed. Fig 6(a) depicts the experimentally measured values of the fiber’s volume fraction *V*_*f*_ in wild moso bamboos. The graph was originally demonstrated by Amada *et al.* in Ref. [17], and we have replicated it using image processing techniques. In Fig 6(a), the measured values of *V*_*f*_ are plotted as a function of the distance from the inner surface of the hollow culm. The index *n* in the legend represents the internode number from the ground; *e.g.,* *n* = 1 at the bottommost internode for a given individual bamboo culm. The plot tells us that in wild moso bamboos, fibers embedded near the bottom of an entire culm distribute quadratically in the radial direction, while those near the top distribute almost linearly.

The characteristic curves of *V*_*f*_ mentioned above are well reproduced using the theory we have developed, as shown in Fig 6(b). In the latter plot, $V_{f}^{\text{inn}}=0.1$ is fixed for all the curves; this definition follows from that $V_{f}^{\text{av}}$ obtained from the experimental data fall within the range of 0.2 to 0.3 [See Fig 6(c)], for which the optimal value of $V_{f}^{\text{inn}}$ equals to 0.1 as was found in Fig 5(a). The set of parameters, (*a*[mm], *h*[mm]), is set to be (50.0, 9.2), (45.0, 8.0), (40.0, 6.7), and (12.5, 3.8) for *n* = 14, 15, 23 and 42, respectively. These values are all estimated from the experimental data shown in Fig 6(a). It is clearly observed that the distribution curves theoretically deduced are in good agreement with the curves obtained through the experiment by Amada *et al.* The agreement between the theory and experiment assures the optimality in the fiber distribution toward reinforcement of elastic hollow cylinders.

## Conclusion

We have determined the optimal design principle for fiber-reinforced cylindrical composites, identical to that realized by wild bamboos. The theory that we have developed predicts that the most effective distribution of reinforcing fibers in the radial direction is to switch from a parabolic to a linear gradation with increasing the mean volume fraction of the fibers. This theoretical consequence matches quantitatively with the real vascular bundle distribution observed in sections of the wild bamboos. It is hoped that our results will open new avenues in the bio-inspired optimal design of cylindrical FGMs endowed with high-stiffness and lightweight properties.

## Supporting information

S1 Fig Photo of bamboo’s cross section. The original graphics data of the bamboo’s cross section presented in the left-hand panel of Fig 1.
(JPG)

S2 Fig Closed-up photo. The original photo data of the enhanced photo shown in the right-hand panel of Fig 1.
(JPG)

S1 File Numeric data. The original numeric data for producing Fig 3a.
(TXT)

S2 File Numeric data. The original numeric data for producing Fig 3b.
(TXT)

S3 File Numeric data. The original numeric data for producing Fig 3c.
(TXT)

S4 File Numeric data. The original numeric data for producing Fig 4.
(TXT)

S5 File Numeric data. The original numeric data for producing Fig 5b.
(TXT)

S6 File Numeric data. The original numeric data for producing Fig 6a.
(TXT)

S7 File Numeric data. The original numeric data for producing Fig 6c.
(TXT)
