# Supplementary figures and images for: Bamboo-inspired optimal design for functionally graded hollow cylinders
Source: PLoS One. 2017 May 3;12(5):e0175029. doi: 10.1371/journal.pone.0175029 (PMC5414991; doi:10.1371/journal.pone.0175029)

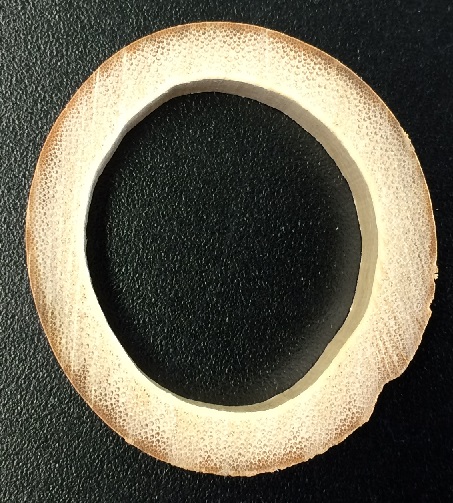

Supplement: S1 Fig — The original graphics data of the bamboo’s cross section presented in the left-hand panel of Fig 1. (JPG) [file pone.0175029.s001.jpg]

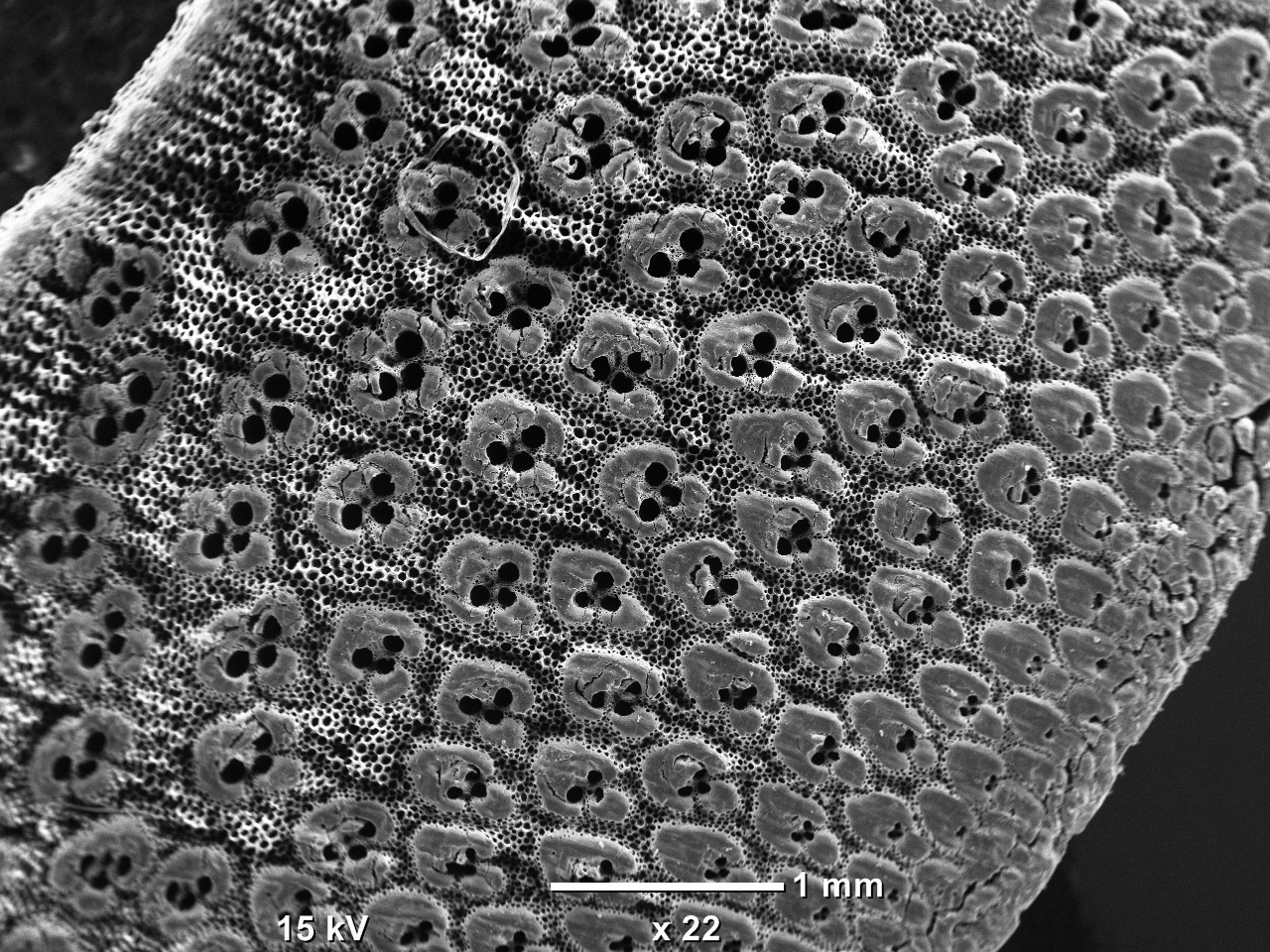

Supplement: S2 Fig — The original photo data of the enhanced photo shown in the right-hand panel of Fig 1. (JPG) [file pone.0175029.s002.jpg]
